# Supplementary material for: Exercise Improves Redox Homeostasis and Mitochondrial Function in White Adipose Tissue
Source: Antioxidants (Basel). 2022 Aug 29;11(9):1689. doi: 10.3390/antiox11091689 (PMC9495527; doi:10.3390/antiox11091689)
Supplement: Supplementary file 1 [file antioxidants-11-01689-s001.zip › antioxidants-1804375-supplementary.pdf]

SUPPLEMENTARY MATERIAL

Supplementary Table S1. F*orward* and r*everse* sequences of primers.

GENE	Acession Number	SPECIES	FOWARD	REVERSE
Nox2	NM_023965.1		*CCATTCACACCATTGCACATC*	*CGAGTCACAGCCACATAC G*
Nox4	NM_053524.1		*TCCATCAAGCCAAGATTCTGAG*	*GGTTTCCAGTCATCCAGTAGAG*
Cat	NM_012520.2		*CAAGCTGGTTAATGCGAATGG*	*TTGAAAAGATCTCGGAGGCC*
Gpx1	NM_030826.4		*AATCAGTTCGGACATCAGGAG*	*GAAGGTAAAGAGCGGGTGAG*
Gpx2	NM_183403.2		*GACACGAGGAAACCGAAGCA*	*GGCCCTTCACAACGTCT*
Gpx3	NM_022525.4		*CAGCTACTGAGGTCTGACAG*	*ACTAGGCAGGATCTCCGAG*
Sod1	NM_017050.1		*TGTGTCCATTGAAGATCGTGTG*	*CTTCCAGCATTTCCAGTCTTTG*
Sod2	NM_017051.2		*GGACAAACCTGAGCCCTAAG*	*CAAAAGACCCAAAGTCACGC*
Sod3	NM_012880.2		*GACCTGGAGATCTGGATGGA*	*GTGGTTGGAGGTGTTCTGCT*
Nef2l2	NM_031789.3		*TTTGTAGATGACCATGAGTCGC*	*TGTCCTGCTGTATGCTGCTT*
Hmox1	NM_012580.2		*ATCGTGCTCGCATGAACACT*	*CAGCTCCTCAAACAGCTCAATG*
Gclm	NM_017305.2		*CAGTGGGCACAGGTAAAACC*	*AATGCAGTCAAATCTGGTGGC*
Gclc1	NM_017305.2	***Rattus norvegicus***	*GGTGACGAGGTGGAGTACAT*	*AACATCGCCGCCATTCAGTA*
Ppargc1a	NM_031347.1		*CACCGCAATTCTCCCTTGTA*	*TGCGGTATTCATCCCTCTTG*
Ppargc1b	NM_176075.3		*TGCCACAACCCAACCAGTCTCA*	*AGCAGTCTCCAGCAGCCCAAAG*
Vegfa	NM_031836.3		*ACCACAGTCCATGCCATCAC*	*TCCACCACCCTGTTGCTGTA*
Srebf1	NM_001276707.1		*GGAGCCATGGATTGCACATT*	*GCTTCCAGAGAGGAGCCCAG*
Ppargc1a	NM_031347.1		*GGTGAAACTCTGGGAGATCCTCC*	*AGCAACCATTGGGTCAGCTCT*
Il1β	NM_031512.2		*GCAATGGTCGGGACATAGTT*	*AGACCTGACTTGGCAGAGGA*
Il6	NM_012589.2		*TCTCTCCGCAAGAGACTTCCA*	*ATACTGGTCTGTTGTGGG*
Tnf	NM_012589.2		*ACCACGCTCTTCTGTCTACTG*	*CTTGGTGGTTTGCTACGAC*
Ccl2	NM_031530.1		*GTGCTGACCCCAATAAGGAA*	*TGAGGTGGTTGTGGAAAAGA*
Cdkn2a	NM_031550.2		*TCCGAGAGGAAGGCGAACTC*	*GCTGCCCTGGCTAGTCTATCTG*
**Cdkn2d**	NM_130812.4		*ACCCCAAGTGAGGGTTTTCT*	*GATCCTCTCTGGCCTCAACA*
Actb	NM_031144.3		*CAC TTTCTACAATGAGCTGCG*	*CTGGATGGCTACGTACATGG*

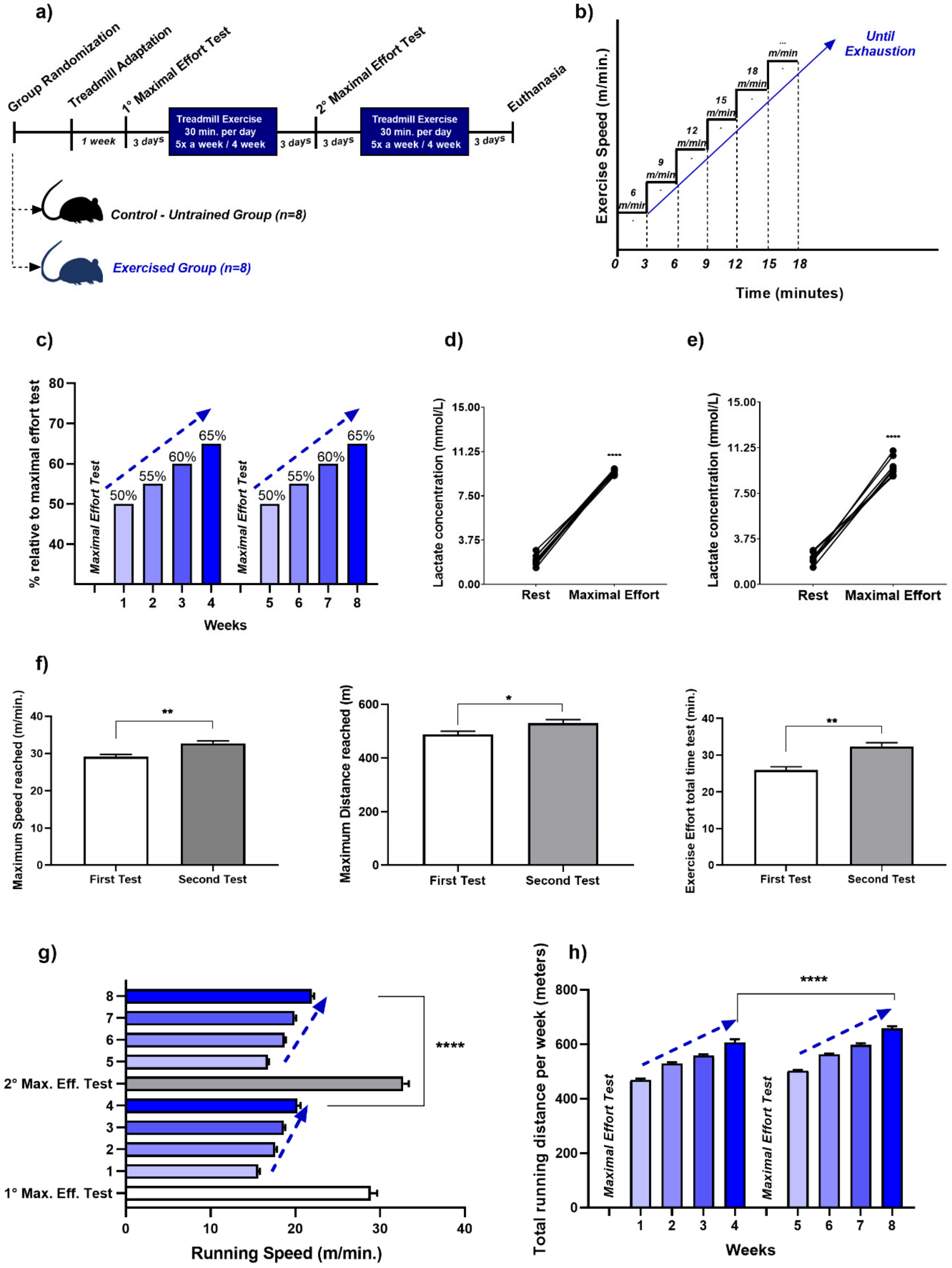

Supplementary Figure S1. Experimental design to aerobic exercise capacity evaluation and periodization of the EX-group. a) Timeline of interventions; b) Graphic representing the maximum speed test protocol; c) Progressive periodization model of aerobic training over the weeks; d) Lactate plasma concentration at rest and maximum post-first and second; (e) maximal speed tests measured in plasma by spectrophotometry using the BioClin® Kit; f) Comparison between the physical performance of animals in the first and last test; g) Maximal speed reached in the 65% of the maximal capacity of AE program; h) Total running distance reached in the 65% of the maximal capacity of AE program. Data were expressed as the mean ± standard error of the mean (n = 8/group). *p<0.05; **p<0.01; ****p <0.0001.
